# Health-related quality of life scores of ultralow rectal cancer patients after conformal sphincter preservation operation compared to newly derived preoperative EORTC QLQ-C30, CR38 reference values and EORTC QLQ-C30 norm

**DOI:** 10.3389/fonc.2026.1724255

**Published:** 2026-02-05

**Authors:** Hai‐bo Ding, Ge Sun, Guan‐yu Yu, Xian‐hua Gao, Zheng Lou, Zheng Kuo, Hai‐feng Gong, Xiao‐ming Zhu, Wei Zhang

**Affiliations:** 1 Department of General Surgery, Yangpu Hospital, School of Medicine, Tongji University, Shanghai, China; 2 Department of Colorectal Surgery, Changhai Hospital, Naval Medical University, Shanghai, China

**Keywords:** conformal sphincter preservation operation, EORTC QLQ-C30, EORTC QLQ-CR38, health-related quality of life, reference values, ultra-low rectal cancer

## Abstract

**Background:**

Postoperative quality of life (Post-QoL) is the key to measuring the effectiveness of sphincter-preserving operation such as Conformal sphincter preservation operation (CSPO) for ultralow rectal cancer. This study aimed to establish preoperative QoL (Pre-QoL) reference values for rectal cancer patients and compare them with post-CSPO QoL to highlight CSPO's benefits in preserving QoL.

**Methods:**

Clinical baseline data and Post-QoL data (EORTC QLQ-C30, CR38) from ultralow rectal cancer patients who underwent CSPO at Shanghai Changhai Hospital from August 2011 to April 2020 were retrospectively collected, and Pre-QoL reference values for rectal cancer patients were extracted through a literature review. The Post-QoL of CSPO were compared with the newly derived Pre-QoL reference values and the norm in the EORTC QLQ-C30 reference value manual.

**Results:**

Compared with the newly derived preoperative C30 reference value, the preoperative C30 norm for colorectal cancer (CRC) and the general population, the Post-QoL of CSPO can exceed the preoperative level and reach a similar level to general population. Compared with the gender, and stage subgroups of the preoperative C30 norm for CRC, the Post-QoL of CSPO showed that male patients benefited more, while patients stage III-IV had limited benefits compared to other subgroups. Compared with the newly derived preoperative CR38 reference value, the Post-QoL of CSPO is comparable to that before surgery, but the sexual function and sexual enjoyment dimensions are significantly lower than those before surgery.

**Conclusions:**

The Long-term QoL after CSPO can exceed the preoperative level and reach a similar level to general population. However, postoperative diarrhea symptoms and decreased sexual function and enjoyment should be taken seriously and measures should be actively taken to promote recovery. Patients stage III-IV had limited benefits compared to other subgroups, and the selection and surgical procedures need to be more cautious.

## 1 Introduction

Owing to the unique anatomical structure of ultralow rectal cancer, preserving anal function and QoL for patients while ensuring oncological safety has always been not only a difficult and hot topic (1) but also a key factor in measuring the success or failure of sphincter-preserving operation (2). In addition, with multimodality therapy becoming the mainstream treatment mode for rectal cancer (3), preoperative and postoperative radiotherapy and chemotherapy can not only improve the anal preservation rate and prognosis but also cause a decline in patients' functional status and QoL (4). Furthermore, studies have shown that QoL is an independent risk factor affecting the prognosis of CRC patients (5). Therefore, the evaluation of QoL after rectal cancer operation, especially after sphincter-preserving operation, is particularly important.

On the basis of the anatomical basis related to anal function after sphincter-preserving operation (6, 7), CSPO have been innovatively proposed by the Colorectal Surgery Department of Shanghai Changhai Hospital (2). CSPO retains more dentate line and distal rectal wall and also protects the autonomic nerve by avoiding dissection in the sphincteric space. In addition, the anastomosis is fashioned on the part with more rectal wall preserved, thus the anastomosis can be 2–3 cm above the dentate line to get more satisfactory anal function after resection. Our center has proven the oncological safety and functional preservation advantages of CSPO over low anterior resection (LAR) and abdominoperineal resection (APR) through previous research (6), and we wrote this technique into the Chinese Expert Consensus on Digestive Tract Reconstruction in Mid-Low Rectal Cancer Surgery (2021 Edition) (8). However, owing to the limitations of retrospective studies, preoperative baseline QoL data are lacking, which makes it difficult to conduct longitudinal comparisons to prove the advantages of CSPO in preserving QoL.

The EORTC QLQ-C30 and CR38 questionnaires (9, 10) are commonly used for QoL evaluation in CRC patients and have been verified to have good reliability and validity in the Chinese population (11, 12). The EORTC QLQ-C30 reference value manual is the only available source of QoL reference values (13), but it has several shortcomings. First, these values come from the combined cohort of CRC patients, which has limited reference value for sphincter-preserving operation. Second, there is a lack of reference value data for the EORTC QLQ-CR38 QoL measurement scale specifically designed for CRC patients. Finally, these reference values have not been updated for over 10 years. However, it is difficult to obtain reference values through standardized preoperative population censuses for rectal cancer (14), while a literature review is more feasible for obtaining QoL data from previously published literature.

Therefore, the purpose of this study was to create a new reference value of QoL for rectal cancer patients through a literature review, take it as the baseline QoL with reference values in the reference value manual, and make a longitudinal comparison with the QoL data after CSPO. To prove the advantages of CSPO in QoL preservation and provide a baseline QoL reference value comparison for future research related to sphincter-preserving operation.

## 2 Method

### 2.1 Study design

We retrospectively collected the clinical baseline data of 123 ultralow rectal cancer patients who underwent CSPO in the Colorectal Surgery Department of Shanghai Changhai Hospital from August 2011 to April 2020. The inclusion and exclusion criteria for patients undergoing CSPO were described previously by Sun et al. (7). The 123 patients were retrospectively followed up with the quality of life questionnaire (EORTC QLQ-C30, CR38). All patients who did not complete the questionnaire were excluded, and all patients who completed the questionnaire were included in the study, regardless of recurrence and metastasis. This study was approved by the Medical Ethics Committee of Changhai Hospital Affiliated with Naval Medical University, approval number B2019-006, and patients and their families signed informed consent forms.

This study consisted of three parts. First, we retrieved and screened literature that included preoperative EORTC QLQ-C30 and CR38 questionnaire data for rectal cancer patients. Second, we obtained new preoperative EORTC QLQ-C30 and CR38 reference values through a literature review. Finally, we compared the postoperative QoL data of CSPO patients with newly derived preoperative reference values, norm values for CRC patients and the general population from the EORTC QLQ-C30 reference value manual.

### 2.2 Main outcome measures

This study applied the EORTC QLQ-C30 and CR38 questionnaires to evaluate QoL in patients with rectal cancer. The QLQ-C30 questionnaire consists of 15 items, including five functional scales (physical function (PF), role function (RF), cognitive function (CF), emotional function (EF), and social function (SF)), three symptom scales (fatigue (FA), pain (PA), nausea/vomiting (NV)), six single-item scales (dyspnea (DY), insomnia (IN), appetite loss (AL), constipation (CO), diarrhea (DI), financial difficulties (FD)), and one global health status scale (GH). The QLQ-CR38 questionnaire consists of 12 items, including four functional scales (body image (BI), sexual function (SF), sexual enjoyment (SE), future perspective (FP)) and eight symptom scales (micturition problem (MP), gastrointestinal symptom (GS), chemotherapy side effect (CSF), stoma problem (SP), male sexual dysfunction (MSD), female sexual dysfunction (FSD), defecation-related problem (DRP), weight loss (WL)). The items in the functional, symptom and single-item scales are scored on a four-point scale (1=not at all, 2=a little, 3=quite a bit, 4=very much), whereas the GH/QoL items are scored on a seven-point scale (from 1=very poor to 7=excellent). All scales are standardized to scores ranging from 0 to 100. Higher scores on the GH/QoL and functional dimensions and lower scores on the symptom dimensions indicate better QoL scores.

These two questionnaires have sufficient reliability, validity and sensitivity (15) and have been widely used internationally. The reliability and validity of the Chinese version have also been validated in Chinese populations Regarding the significance of changes in QoL scores, a mean score change of 5–10 points was reported as "a little" change, 10–20 points was reported as "moderate" change, and >20 points was reported as "very much" change (16, 17).

### 2.3 Extraction of new preoperative EORTC QLQ-C30 and CR38 reference values for rectal cancer patients

#### 2.3.1 Literature search

We searched for literature on the EORTC QLQ-C30 and CR38 questionnaires for rectal cancer patients published in the PubMed, Embase, Web of Science, and Cochrane Library databases until December 21, 2022. The search strategies are shown in **Table 1**.

**Table 1:** Search strategies of each database.

Questionnaire	Database	Search<ns/>	Strategy
EORTC QLQ-C30	PubMed, Embase, Web of Science, Cochrane library databases	1	Disease terms: exp. ((((((((((((((((((Neoplasm, Rectal[Title/Abstract]) OR (Rectal Neoplasm[Title/Abstract])) OR (Rectum Neoplasms[Title/Abstract])) OR (Neoplasm, Rectum[Title/Abstract])) OR (Neoplasm, Rectum[Title/Abstract])) OR (Rectum Neoplasm[Title/Abstract])) OR (Rectal Tumors[Title/Abstract])) OR (Rectal Tumor[Title/Abstract])) OR (Tumor, Rectal[Title/Abstract])) OR (Neoplasms, Rectal[Title/Abstract])) OR (Cancer of Rectum[Title/Abstract])) OR (Rectum Cancers[Title/Abstract])) OR (Rectal Cancer[Title/Abstract])) OR (Cancer, Rectal[Title/Abstract])) OR (Rectal Cancers[Title/Abstract])) OR (Rectum Cancer[Title/Abstract])) OR (Cancer, Rectum[Title/Abstract])) OR (Cancer of the Rectum[Title/Abstract]))).ab,ti.
		2	Questionnaire terms: ((((((((European Organization for Research and Treatment of Cancer quality of life questionnaire[Title/Abstract]) OR (EORTC QLQ-C30[Title/Abstract])) OR (EORTC QLQ C30[Title/Abstract])) OR (EORTC C30[Title/Abstract])) OR (EORTC-C30[Title/Abstract])) OR (QLQ-C30[Title/Abstract])) OR (QLQ C30[Title/Abstract])) OR (C30[Title/Abstract])).ab,ti.
		3	<ns/>1 AND <ns/> 2
EORTC QLQ-CR38	PubMed, Embase, Web of Science, Cochrane library databases	1	Disease terms: exp. ((((((((((((((((((Neoplasm, Rectal[Title/Abstract]) OR (Rectal Neoplasm[Title/Abstract])) OR (Rectum Neoplasms[Title/Abstract])) OR (Neoplasm, Rectum[Title/Abstract])) OR (Neoplasm, Rectum[Title/Abstract])) OR (Rectum Neoplasm[Title/Abstract])) OR (Rectal Tumors[Title/Abstract])) OR (Rectal Tumor[Title/Abstract])) OR (Tumor, Rectal[Title/Abstract])) OR (Neoplasms, Rectal[Title/Abstract])) OR (Cancer of Rectum[Title/Abstract])) OR (Rectum Cancers[Title/Abstract])) OR (Rectal Cancer[Title/Abstract])) OR (Cancer, Rectal[Title/Abstract])) OR (Rectal Cancers[Title/Abstract])) OR (Rectum Cancer[Title/Abstract])) OR (Cancer, Rectum[Title/Abstract])) OR (Cancer of the Rectum[Title/Abstract]))).ab,ti.
		2	Questionnaire terms: ((((((((European Organization for Research and Treatment of Cancer quality of life questionnaire[Title/Abstract]) OR (EORTC QLQ-CR38[Title/Abstract])) OR (EORTC QLQ CR38[Title/Abstract])) OR (EORTC C30[Title/Abstract])) OR (EORTC-C30[Title/Abstract])) OR (QLQ-C30[Title/Abstract])) OR (QLQ C30[Title/Abstract])) OR (C30[Title/Abstract])).ab,ti.
		3	<ns/>1 AND <ns/> 2

#### 2.3.2 Inclusion criteria

- Original research articles published in international journals;
- Adequate sample size (≥ 20 cases) for each literature;
- The research subjects were clinically diagnosed with rectal cancer;
- Literature directly reported the mean and standard deviation of each dimension of the preoperative EORTC QLQ-C30 and CR38 questionnaires, or we could obtain them through data calculation or extraction from the literature charts;
- Moderate to high quality literature.

#### 2.3.3 Exclusion criteria

- Reviews, animal research, basic research, case reports, research protocols, conference papers, etc.
- Studies that did not report the mean values and standard deviations of each dimension of the preoperative EORTC QLQ-C30 and CR38 questionnaires or had more than 20% missing data.
- Duplicate publications or studies.

#### 2.3.4 Literature screening and quality assessment

The literature retrieval was conducted by two independent researchers according to a unified search strategy. NoteExpress literature management software was used to screen all the retrieved literature. For literature which was uncertain whether they could be included, two researchers discussed them first. If they could still not decide, a third researcher intervened to decide whether to include it. If they could still not decide, they contacted the author to decide whether to include it.

The quality of the included literature was assessed via the Cochrane Risk of Bias Tool for randomized controlled trials (18), the Newcastle–Ottawa Scale for case–control studies (19), and the Agency for Healthcare Research and Quality cross-sectional study evaluation criteria for cross-sectional studies (20).

#### 2.3.5 Data extraction and summarization

The sample size, source of patients, research methods, authors, and year of all included literature were summarized, the mean and standard deviation of each dimension were extracted from the preoperative EORTC QLQ-C30 and CR38 questionnaires of all included literature, and weighted averaging was performed to obtain a new reference value.

#### 2.3.6 Assessment of heterogeneity

Given the high clinical and methodological heterogeneity of patient populations, regions, and treatment backgrounds in the study. We plan to use the I^2^ statistic and Cochran's Q test to preliminarily quantify the heterogeneity caused by regional differences in studys. In addition, we will report the 95% confidence interval for each dimension to more comprehensively reflect the distribution range of the reference values (21).

### 2.4 Comparative study of the post-QoL of CSPO patients

The new reference values for the Pre-QoL of rectal cancer patients and the CRC patient norm in the EORTC QLQ-C30 reference value manual can serve as baseline values for the Pre-QoL of rectal cancer patients. The general population norm of the EORTC QLQ-C30 reference value manual can be used as a control for the QoL of general population. The Post-QoL of retrospectively collected CSPO patients was compared with the above reference values.

### 2.5 Statistical analysis

SPSS 26.0 was used for data analysis. The QoL data were expressed as X ± S. A simple t-test was used for comparisons between groups. P<0.05 indicated a statistically significant difference. A difference in the QoL score of >10 points was considered to indicate a clinically significant difference.

## 3 Results

### 3.1 Literature review

**Figure 1:**
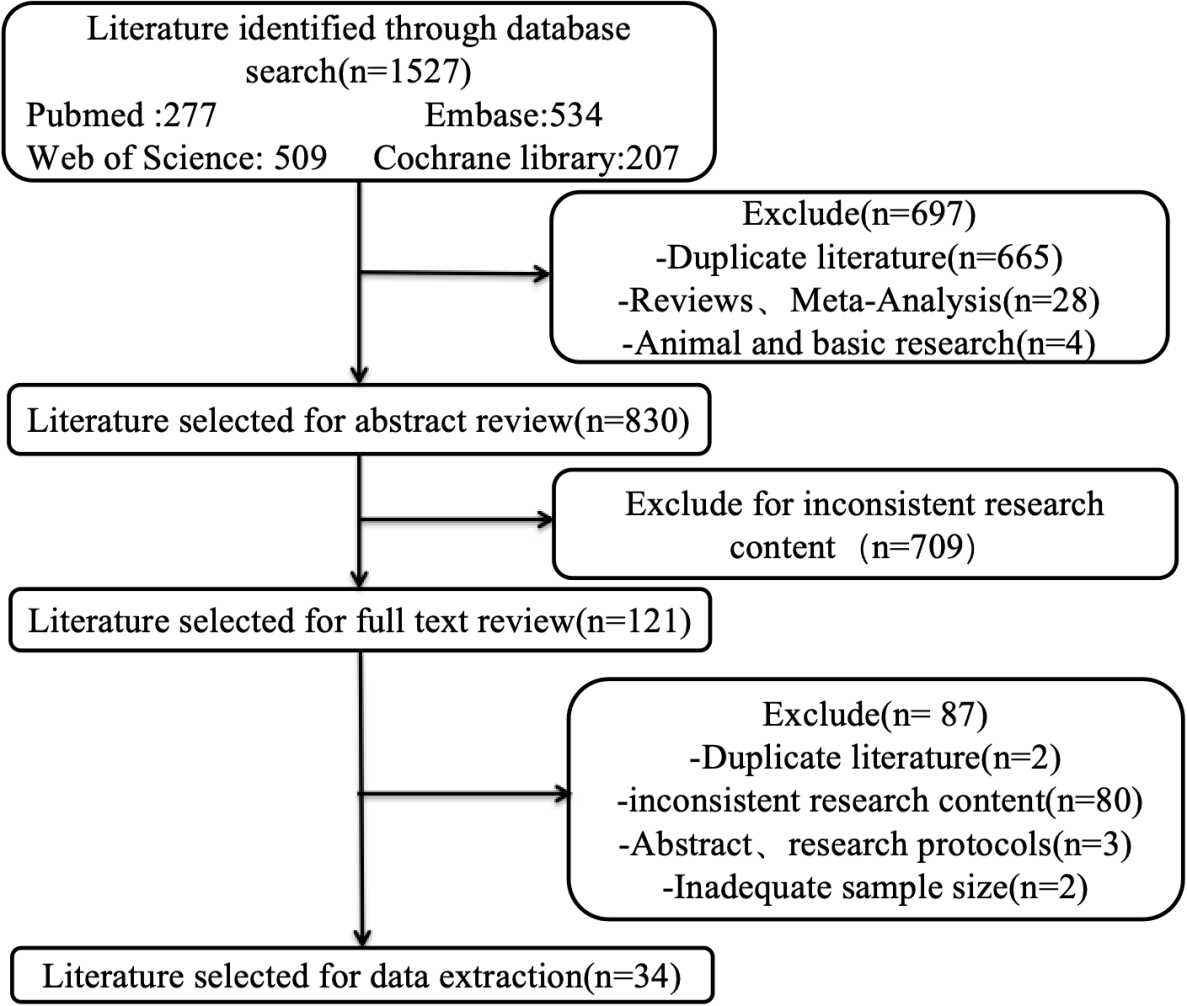
The literature screening flow chart of EORTC QLQ-C30.

A total of 1527 literature that used the EORTC QLQ-C30 were retrieved, and 34 were ultimately included after screening (**Figure 1**). The included literature involved 7117 patients, mainly from China, Germany, the Netherlands, the UK, Australia, Korea, France and other countries, and all were moderate to high quality researches (**Supplementary Tables 1**-**3**).

**Figure 2:**
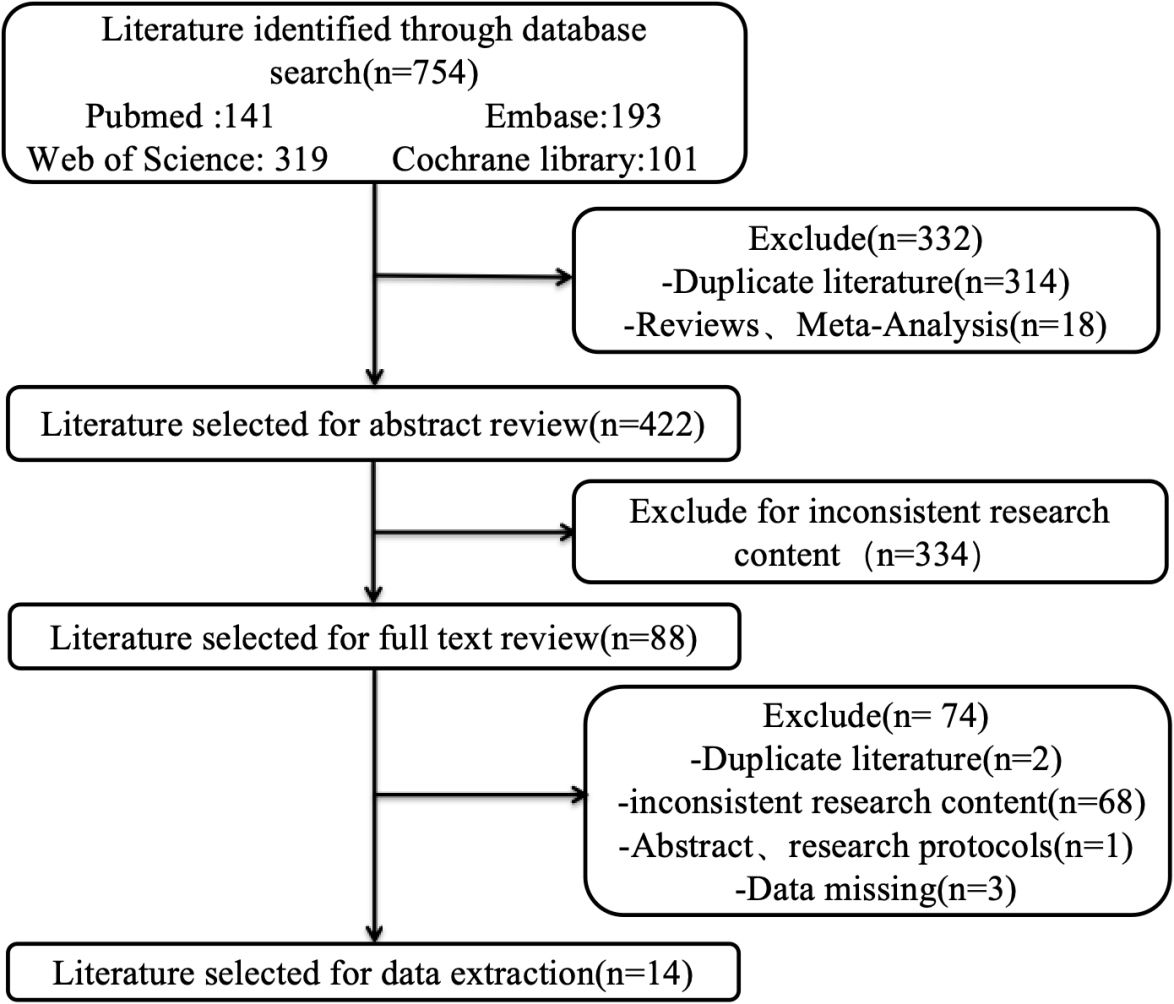
The literature screening flow chart of EORTC QLQ-CR38.

A total of 754 literature that used the EORTC QLQ-CR38 were retrieved, and 14 articles were ultimately included in the quality assessment (**Figure 2**). The included articles involved 3925 patients, mainly from China, Germany, Korea, Australia, Japan and other countries, and all were moderate to high quality researches (**Supplementary Tables 1**-**3**).

The basic information of the included literature is shown in **Table 2**.

**Table 2:** The basic information of the included literature.

Author	Year	n	Country	Questionnaires	
Simon et al.	2013	74	China	C30	CR38
Kang et al.	2020	342	Korea	C30	CR38
Gilbert et al.	2022	88	UK	C30	
Zheng et al.	2018	171	China	C30	CR38
Irene et al.	2022	389	German	C30	CR38
Zhang et al.	2015	852	China	C30	CR38
Morielli et al.	2021	32	Canada	C30	
Andersson et al.	2013	385	Belgium, Canada, Denmark, German, the Netherlands, Korea, Spain, Sweden	C30	CR38
Sun et al.	2018	220	China	C30	
Rodrigo et al.	2021	61	Brazil	C30	CR38
Rebekka et al.	2021	1025	German	C30	CR38
Laetitia et al.	2008	306	France	C30	
Francesco et al.	2012	164	UK, Spain, Sweden	C30	
McLachlan et al.	2016	297	Australia	C30	CR38
Yumiko et al.	2017	137	Japan	C30	CR38
Alice et al.	2017	272	the Netherlands	C30	
Hompes et al.	2014	92	UK	C30	
Lacy et al.	2022	45	Spain	C30	
Schmidt et al.	2005	368	German	C30	
Alves et al.	2018	29	Brazil	C30	CR38
Stephanie et al.	2019	51	Australia	C30	
Hosni et al.	2022	101	Canada	C30	
Bencova et al.	2016	64	Slovakia	C30	
Vaneja et al.	2020	61	Slovenia	C30	
Anna et al.	2022	223	Denmark	C30	
Kurian et al.	2016	54	Canada	C30	
Tjandra et al.	2001	42	Australia	C30	
Han et al.	2014	54	Canada	C30	
Schmidt et al.	2005	58	German	C30	
Juan et al.	2006	83	Spain	C30	CR38
Herman et al.	2012	50	America	C30	CR38
Qaderi et al.	2021	710	the Netherlands	C30	
Anneke et al.	2013	30	Canada	C30	CR38
Gilbert et al.	2020	187	UK	C30	

### 3.2 Data summary

The newly derived reference values are summarized in **Table 3**, **4**. 105 of the 123 CSPO patients completed the EORTC QLQ-CR30 and CR38 questionnaires and were included in the study, with a response rate of 85.37%. The median follow-up time for CSPO patients was 35 (7–114) months. The baseline characteristics of CSPO patients are summarized in **Table 5**, and the demographic baseline data comparison between CSPO and reference values is shown in **Table 6**. The results of heterogeneity analysis in different regions of patients are shown in **Supplementary Table 4**.

**Table 3:** Newly derived reference values of preoperative EORTC QLQ-C30 for rectal cancer patients.

Domain	(PF) Physical function	(RF) Role function	(EF) Emotional function	(CF) Cognitive function	(SF) Social function	(GH) Global health status	(FA) Fatigue	(NV) Nausea/vomiting	(DY) Dyspnea	(PA) Pain	(IN) Insomnia	(AL) Appetite loss	(CO) Constipation	(DI) Diarrhea	(FD) Financial difficulties
N	7117	7117	7117	7117	7117	7117	7117	7117	7117	7117	7117	7117	7117	7117	6778
Average	86.4	79.8	73.5	84.9	78.9	66.8	26.0	5.3	10.7	19.5	27.1	13.7	16.4	21.7	15.5
SD	17.5	27.4	22.8	19.9	24.6	22.3	24.3	16.0	21.9	26.2	29.6	25.0	26.9	28.9	27.4
95%CI	51.5-100	25-100	27.9-100	45.1-100	29.8-100	22.1-100	0-74.6	0-37.3	0-54.5	0-71.9	0-86.4	0-63.7	0-70.2	0-79.5	0-70.2

**Table 4:** Newly derived reference values of preoperative EORTC QLQ-CR38 for rectal cancer patients.

Domain	(BI) Body image	(SF) Sexual function	(SE) Sexual enjoyment	(FP) Future perspective	(MP) micturition problem	(CSF) Chemotherapy side effects	(GS) Gastrointestinal symptoms	(MSD) Male sexual dysfunction	(FSD) Female sexual dysfunction	(DRP) Defecation-related problem	(WL) Weight loss
N	3925	3540	1744	3925	3540	3759	3925	2769	2389	3194	3536
Average	82.9	42.2	53.0	53.0	23.8	12.8	17.5	36.2	22.2	25.6	17.5
SD	23.5	33.6	30.0	31.2	17.7	15.6	16.4	33.1	26.9	18.5	26.2
95%CI	35.8-100	0-100	0-100	0-100	0-59.1	0-43.9	0-50.2	0-100	0-75.9	0-62.6	0-69.9

**Table 5:** Baseline characteristics of CSPO.

	Total (n=105)
Gender (cases)	
Male	67 (64%)
Female	38 (36%)
Age (years)	60.86 ± 10.42
BMI (Kg/m²)	22.78 ± 3.18
Neoadjuvant therapy	
Yes	37 (35%)
No	68 (65%)
Distance of tumor from anal verge (cm)	3.5 ± 1.1
cTNM staging	
I-II	47 (45%)
III-IV	58 (56%)
pTNM staging	
I-II	85 (81%)
III-IV	20 (19%)
Postoperative complications	
Yes	18 (17%)
No	87 (83%)
Clavien–Dindo complications classification	
0	87 (83%)
I	3 (3%)
II	14 (13%)
III	1 (1%)
IV	0
Postoperative hospital stay (days)	6.5 ± 2.5
Closure of stoma	
Yes	100 (95%)
No	5 (5%)
Duration of stoma (Median months)	8
PFS3	92/105; 88%
OS3	102/105; 97%

**Table 6:** The demographic baseline data comparison between CSPO and reference values.

Questionnaires	Gender		*P*	Age				*P*	TNM stage		*P*
	Male	Female		Average	N	Standard error	N		I-II	III-IV	
CSPO	67	38		60.86	105	10.42	105		47	58	
C30	4158	2876	0.331	61.12	4430	10.6	2222	0.804	2710	3444	0.882
CR38	2450	1392	0.993	62.46	1234	10.8	887	0.144	1510	1910	0.901
C30 reference value	13225	9028	0.362						4720	8066	0.097
CSPO male subgroup									30	37	
C30 male subgroup									2705	4910	0.115
CSPO female subgroup									16	22	
C30 female subgroup									1669	2483	0.811
CSPOI-II stage	31	16									
C30 I-II stage	2705	1669	0.563								
CSPOIII-IV stage	36	22									
C30 III-IV stage	4910	2483	0.485								

### 3.3 Comparison between the CSPO postoperative C30 score and the newly derived preoperative C30 reference value

**Figure 3:**
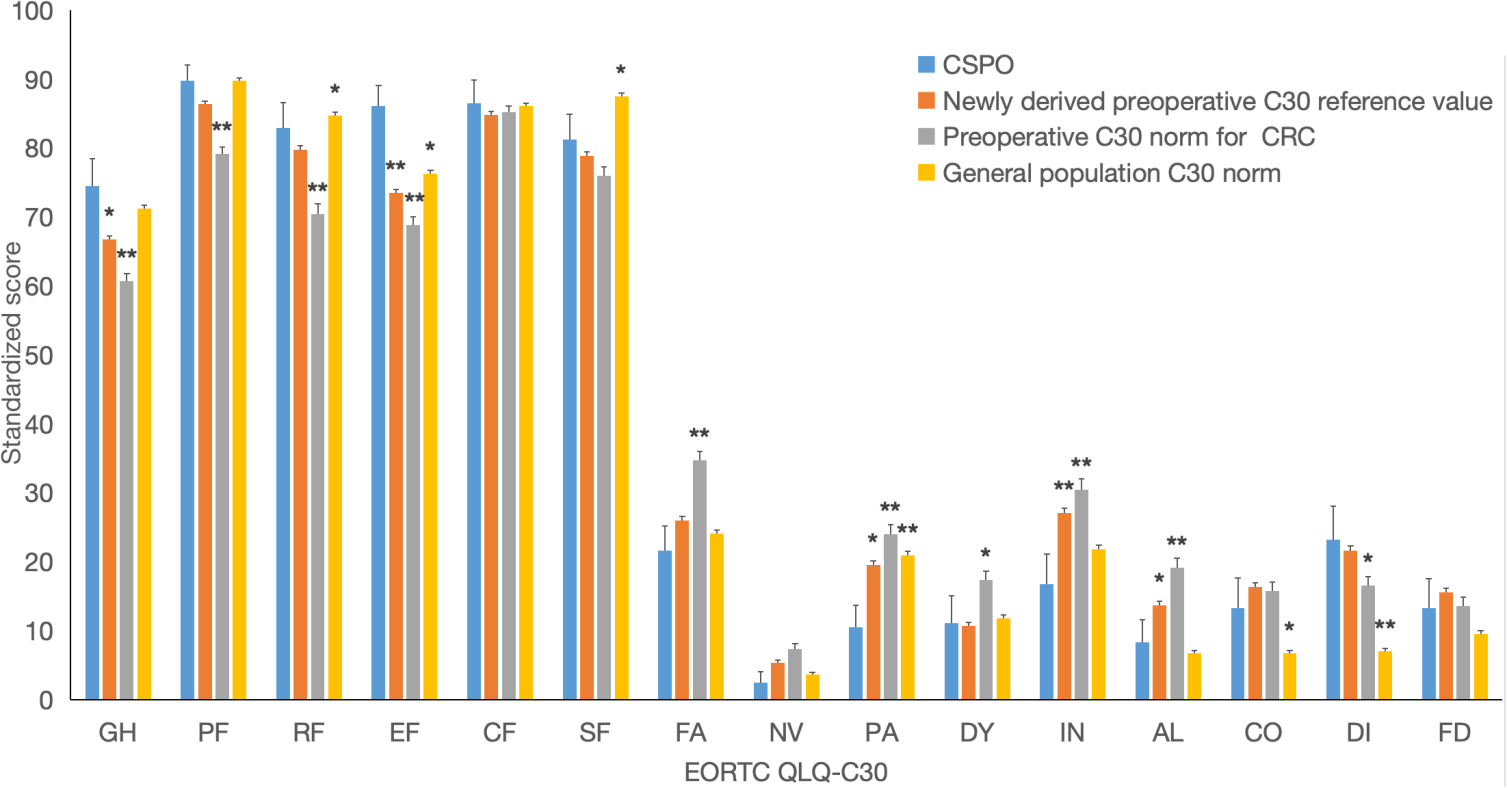
Comparison of postoperative C30 score of CSPO with newly derived preoperative C30 reference value and C30 norm. * indicates a significant difference and a difference 5, ** indicates a significant difference and a difference 10.

Compared with the newly derived preoperative C30 reference value for rectal cancer patients, there was no statistically significant difference in demographic baseline between CSPO and reference value. However, CSPO patients showed statistically significant differences in Post-QoL in 5 out of 15 dimensions of the C30 scale, and all of those patients had better QoL scores. Among them, the GH was 7.7 points higher, and there were significant differences in the EF dimension (12.6 points higher) and symptom dimension (IN lower by more than 10 points; PA and AL lower by more than 5 points). There was a significant difference in the PF dimension, but it was not clinically significant (3.4). These results indicate that with a median follow-up time of 35 months, the Post-QoL of CSPO patients exceeded the Pre-QoL of rectal cancer patients. (**Figure 3**, error bars with 95% CI, * indicates a significant difference and a difference ≥ 5, * * indicates a significant difference and a difference ≥ 10, * * * indicates a significant difference and a difference ≥ 20).

### 3.4 Comparison of the CSPO postoperative C30 score and preoperative C30 norm in patients with CRC

Compared with the preoperative C30 norm of the CRC, there was no statistically significant difference in demographic baseline between CSPO and C30 norm. However, CSPO patients showed statistically significant differences and better QoL scores in 10 out of 15 dimensions of the C30 scale. Among them, the GH was 13.8 points higher, and there were significant differences in functional dimensions (PF, RF), and EF were all more than 10 points higher) and symptom dimensions (FA, PA, IN, and AL were more than 10 points lower, and DY dimensions were more than 5 points lower). There was a statistically significant difference in NV symptoms, but the difference was not significant (-4.8); however, DI (6.6) symptoms were slightly improved in the CSPO group. This finding indicates that with a median follow-up time of 35 months, the Post-QoL of CSPO patients can significantly exceed the Pre-QoL of CRC patients. (**Figure 3**).

### 3.5 Comparison between the CSPO postoperative C30 score and the general population C30 norm

Compared with the general population C30 norm, there was no significant or clinical difference in the Post-QoL of CSPO patients in 11 out of 15 dimensions of the C30 scale. CSPO patients have a mild decrease in SF (-6.2) and a moderate improvement in DI symptoms (16.2) but a mild improvement in EF (9.8) and a moderate decrease in PA symptoms (-10.4). This finding indicates that with a median follow-up time of 35 months, the Post-QoL of CSPO patients can reach a level similar to that of the general population. (**Figure 3**).

### 3.6 Comparison of gender subgroups between the CSPO postoperative C30 score and preoperative C30 norm in patients with CRC

**Figure 4:**
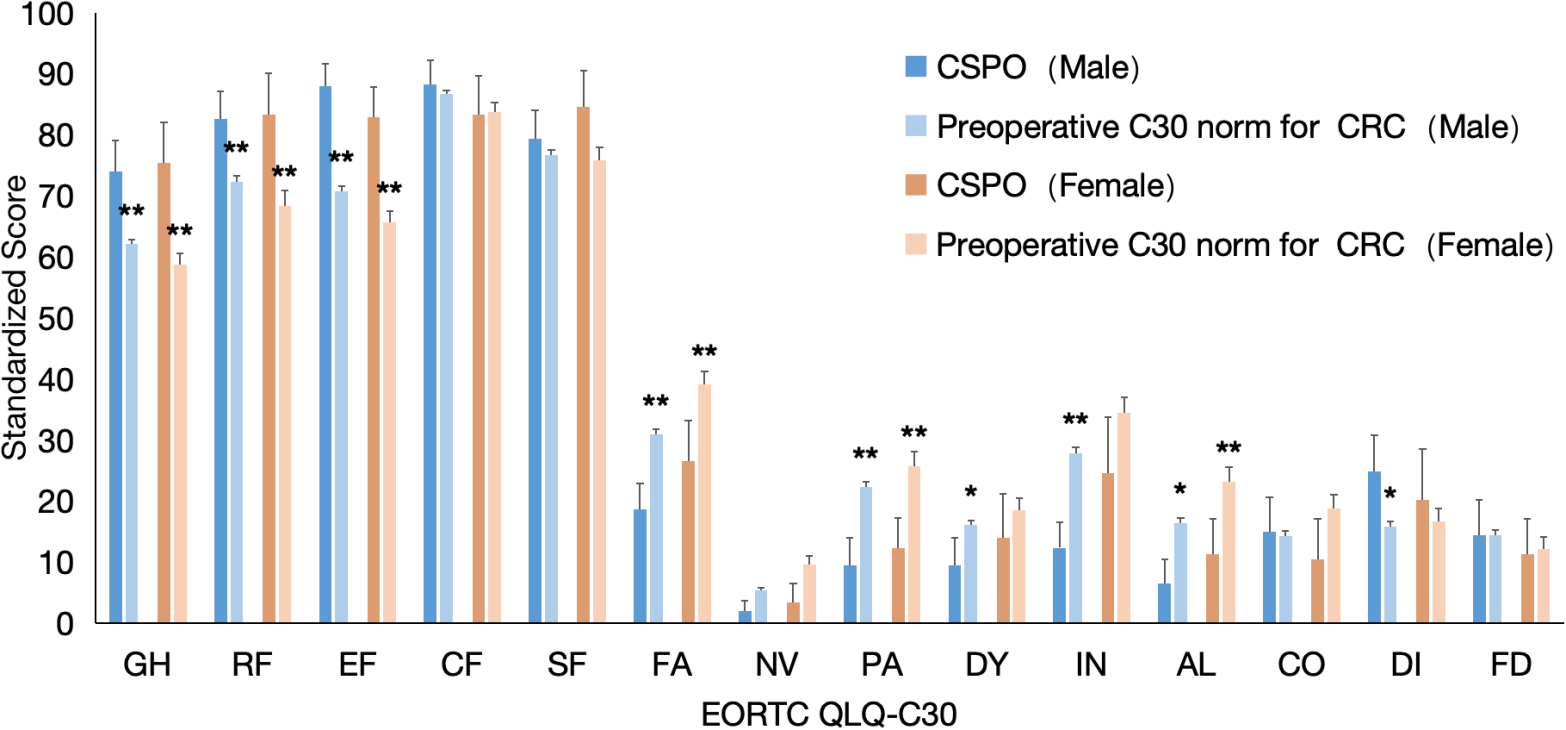
Comparison of gender subgroups between the CSPO postoperative C30 score and preoperative C30 norm in patients with CRC. * indicates a significant difference and a difference 5, ** indicates a significant difference and a difference 10.

Compared with the preoperative C30 norm of the CRC, regardless of gender, the CSPO showed statistically significant differences in GH, functional dimensions (PF, RF, EF), and symptom dimensions (AL, FA, PA), with better scores ranging from mild to moderate. There was no statistically significant difference in demographic data between the CSPO and preoperative C30 norm gender subgroups. In addition, compared with the female subgroup, the male subgroup presented a moderate decrease in the scores of DY and IN, but at the same time, the male subgroup presented a slight improvement in the DI (9.1). On the basis of the above analysis results, the score differences in the dimensions of DY, IN, and DI in the CSPO group are attributed mainly to the male subgroup. Compared with the female subgroup, the male subgroup benefits more from the Post-QoL of CSPO patients. (**Figures 4**).

### 3.7 Comparison of stage subgroups between the CSPO postoperative C30 score and preoperative C30 norm in patients with CRC

**Figure 5:**
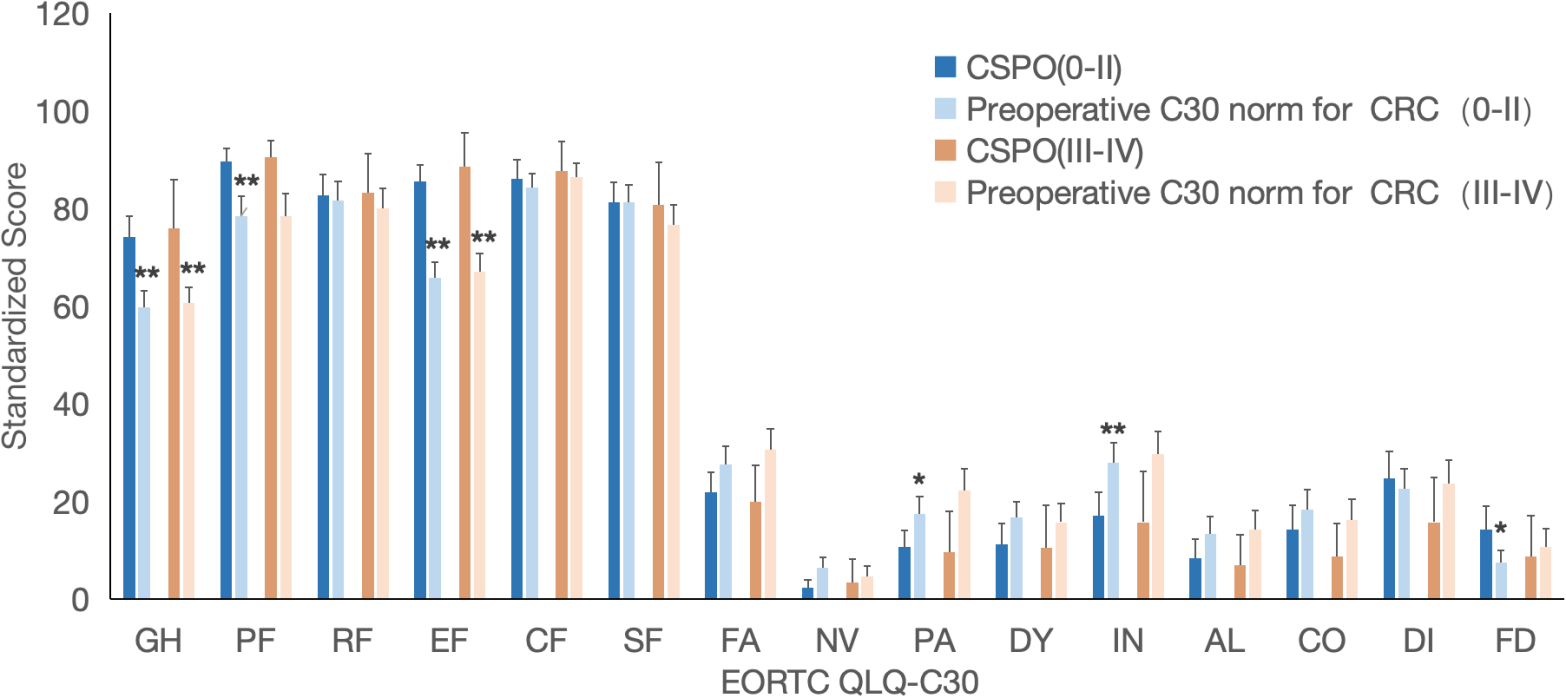
Comparison of stage subgroups between the CSPO postoperative C30 score and preoperative C30 norm in patients with CRC. * indicates a significant difference and a difference 5, ** indicates a significant difference and a difference 10.

There was no statistically significant difference in demographic data between the CSPO and preoperative C30 norm stage subgroups. In addition, compared with the preoperative C30 norm of CRC patients, the GH, functional dimensions (PF, EF), and symptom dimensions (IN, PA) of the 0-II stage subgroup in CSPO patients had mildly to moderately better QoL scores than the preoperative levels did, but there was a slight increase in the FD dimension (6.7). In the III-IV stage subgroup, there was only a moderate improvement in GH (15.2) and a significant improvement in EF (21.5). (**Figures 5**).

### 3.8 Comparison between the CSPO postoperative CR38 score and the newly derived preoperative CR38 reference value

**Figure 6:**
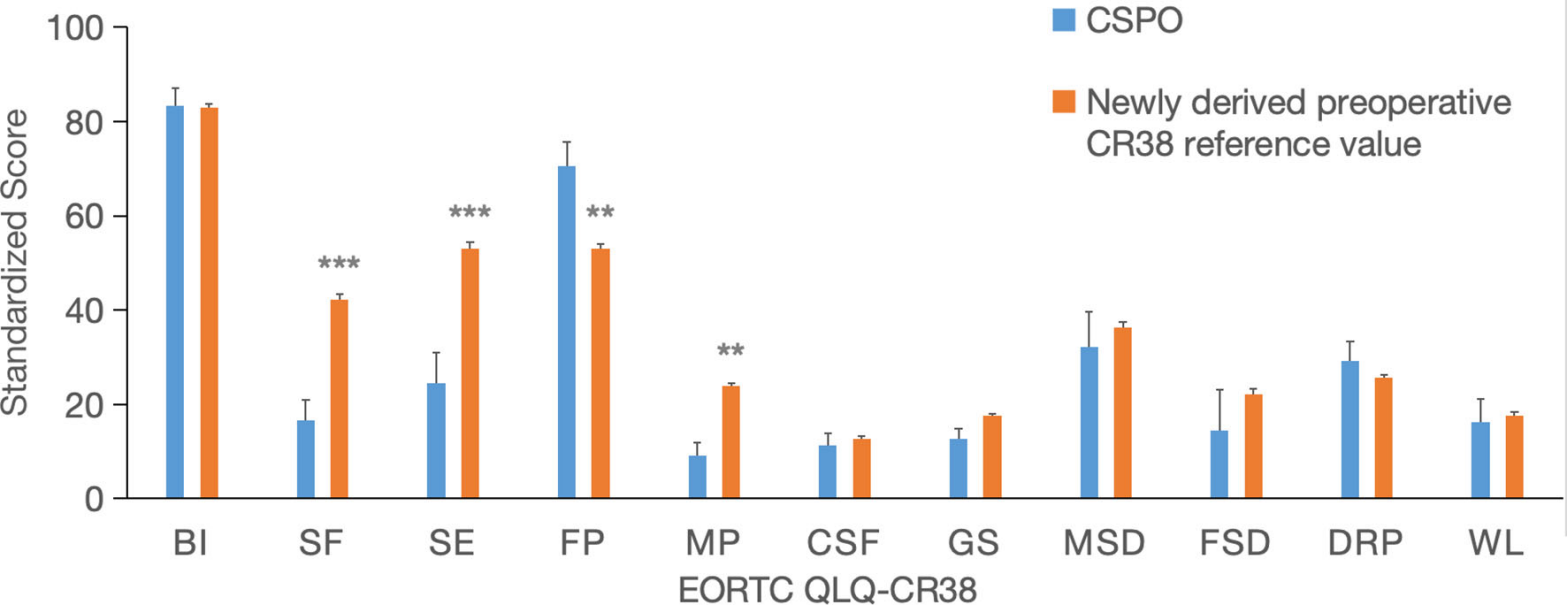
Comparison between the CSPO postoperative CR38 score and the newly derived preoperative CR38 reference value. ** indicates a significant difference and a difference 10, *** indicates a significant difference and a difference 20.

Compared with the newly derived preoperative CR38 reference value for rectal cancer patients, there was no statistically significant difference in demographic baseline between CSPO and reference value. However, CSPO patients showed a significant decrease in the SF (–26) and SE (–29) dimensions but a moderate improvement in the FP (17.5) and a moderate decrease in the MP (–15). (**Figures 6**).

## 4 Discussion

This is the first study to systematically extract preoperative EORTC QLQ-C30 and CR38 reference values for rectal cancer patients through a literature review. The reference values obtained reflect the QoL of rectal cancer patients at the time of diagnosis.

The reference values obtained are mainly from published studies with high heterogeneity, which have certain limitations. However, this study provides the most comprehensive and quantifiable approximate reference range so far, reflecting the quality of life of patients with rectal cancer at the time of diagnosis. These reference values can be used as baseline data supplements for retrospective studies of rectal cancer surgery to evaluate the impact of various treatment interventions on QoL. In addition, when clinical studies are conducted with Post-QoL as the outcome for rectal cancer patients, it can also be used as a baseline reference for QoL, which is helpful for the development and analysis of these studies (14, 22).

Compared with intersphincteric resection (ISR), CSPO not only protects the autonomic nerves in the sphincter gap but also retains more of the contralateral intestinal tube, which theoretically is conducive to protecting the patient's anal function and improving Post-QoL. As expected, compared with the preoperative baseline QoL (newly derived preoperative C30 reference value and preoperative C30 norm of CRC) and the general population (general population C30 norm), the study revealed that low rectal cancer patients who underwent CSPO had significantly greater QoL than the preoperative level at a median follow-up time of 35 months and reached a level similar to that of the general population. This finding is consistent with the results of Pappou et al., who reported that the QoL of postoperative rectal cancer patients showed a "V"-shaped change, with the lowest level occurring at 6 and 12 months after surgery, and gradually recovered or even exceeded the preoperative level at 24 months (23).

Horizontal comparison with three reference values revealed that the global health status and functional dimensions of CSPO patients improved after surgery, but their social function was still mildly impaired compared with that of the healthy population, which might be related to anal dysfunction. After sphincter-preserving operation, some patients may experience low anterior resection syndrome (LARS) such as urgency, frequency, and fecal incontinence, which persist for several years and seriously affect their daily life and social activities (24). In addition, postoperative constipation and diarrhea symptoms in CSPO patients increased to varying degrees compared with preoperative levels, especially compared with those in the general population and CRC population, whereas there was no significant difference compared with the newly derived preoperative reference value for rectal cancer. This first indicated that postoperative constipation and diarrhea symptoms persist for a long time and reduce QoL, which is also consistent with a decline in social functioning. Second, constipation and diarrhea symptoms were more obvious in rectal cancer patients than in the general population and CRC population. This finding was also consistent with the results of Chen et al., who reported that LARS symptoms persist and affect the Post-QoL of some rectal cancer patients (25), which indirectly suggests that we should pay more attention to the prevention and treatment of postoperative constipation and diarrhea symptoms in rectal cancer patients (26).

Compared with the preoperative gender subgroup reference values of CRC, the male subgroup had more significant improvements in dyspnea and insomnia symptoms than the female subgroup did after CSPO, and the overall decrease in scores in these two symptom dimensions was attributed mainly to the male subgroup. However, the male subgroup also has more diarrhea symptoms after CSPO, and the overall improvement in scores in this symptom dimension in the CRC population is attributed mainly to the male subgroup. This reflects the impact of gender differences on physical and psychological aspects of QoL. Due to the different pelvic anatomy of women and other accompanying factors such as obstetric trauma and pelvic floor dysfunction, it may further affect their QoL (27) and should be taken seriously by doctors. In addition, the diarrhea in the male subgroup after CSPO may be attributed to the narrowness of the pelvic cavity and the lack of radiation protection for the rectum by organs such as the uterus, resulting in increased radiation doses. On the other hand, this may be attributed to the inhibition of intestinal endothelial cell function by androgens, resulting in slow recovery of intestinal function after radiotherapy (28).

Compared with the preoperative tumor stage subgroup reference values of CRC patients, stage III-IV patients had limited QoL benefits compared with stage 0-II patients after CSPO (only moderate improvement in global health status and significant improvement in emotional function). This may be related to various factors of advanced rectal cancer, such as a higher proportion of patients with radiochemotherapy, more tissue being resected to ensure radical resection, more severe postoperative complications, shorter expected survival time, and heavier economic burden (29, 30). This finding is consistent with the results of Han et al., who reported that advanced tumor stage is a risk factor for poor QoL in postoperative CRC patients (31). This finding suggests that for patients in advanced tumor stage, the QoL benefits of sphincter-preserving operation may be limited, so the selection and surgical operation of sphincter-preserving operation need to be more cautious.

Compared with the newly derived preoperative CR38 reference value for rectal cancer, CSPO showed a moderate increase in the future perspective dimension and a moderate decrease in the micturition problem dimension. However, the postoperative sexual function and sexual enjoyment dimensions were significantly lower in the CSPO group than in the control group, and there was no difference in the male or female sexual dysfunction dimensions between the two groups. These findings indicate that CSPO provides sufficient protection for the pelvic nerve plexus in patients and that there is no significant increase in postoperative sexual dysfunction. The significant decrease in the sexual function and sexual enjoyment of patients may be related to several factors. First, after CSPO, prophylactic ileostomy is routinely performed, which is then reversed 3–6 months later. After ileostomy reversal, patients need to adapt to the new rectum and experience a period of anal dysfunction, which puts them in a difficult stage for a long time, greatly affecting their interest and pleasure in sexual life (32). Second, unlike the Western population, many couples in China have no sex life after the age of 50 years due to the influence of traditional habits (33), and the answers to sexual questions are relatively conservative, which affects the results (34). Finally, both the CSPO population and the reference population have relatively low response rates to several items related to sexual function, which may not reflect the true situation. This suggests that for patients who plan to undergo CSPO, doctors should fully inform them of the negative effects on postoperative sexual function and pleasure before surgery. In addition, postoperative measures such as early ileostomy reversal, anal lifting exercise, and neurophysiological treatment should be actively taken to shorten the process of anal dysfunction (35).

This study is the first to explore the reference values of the EORTC QLQ-C30 and CR38 through a literature review, but it has several limitations. First, the 43 articles included in this study had significant differences in population characteristics, geographic regions, measurement methods, etc., resulting in high heterogeneity when data were merged. In addition, no special postoperative bowel-function instruments were used to evaluate bowel dysfunction, and the impact of various factors on postoperative anal function and quality of life was only analyzed through univariate comparisons, without considering the influence of confounding factors. These may not only affect the validity of the reference values, but the interpretation of related conclusions also needs to be more cautious. Second, the QoL reference values in this study were obtained from rectal cancer patients who had not received treatment after diagnosis. There is a lack of data on patients with stable conditions after neoadjuvant therapy, and the reference values of these patients may be more suitable for comparative studies of sphincter-preserving operation. Third, we adopted quite strict inclusion criteria, but owing to the large sample size, this does not affect the robustness of the reference values. In addition, due to the exclusion of patients who were lost to follow-up after CSPO, this selection bias may affect the reliability of the results. However, our overall questionnaire completion rate was 85.37%, and this impact is also limited. Fourth, in the comparative study between CSPO and various reference values, we compared the available demographic baseline data as much as possible to eliminate baseline differences. However, the available demographic data is limited, so it may not be possible to completely eliminate inter group differences that could affect the reliability of the results. In addition, when comparing the age subgroups of CSPO and C30 reference values, there were differences in baseline between the two groups due to factors such as too many subgroups, so further analysis and comparison were not conducted. For these reasons, it is necessary in the future to establish a more reliable and clinically comparable QoL reference value system by implementing standardized data collection, subgroup stratification, and multidimensional assessment.

In summary, this study revealed that low rectal cancer patients who underwent CSPO had a significant improvement in QoL after a median follow-up time of 35 months and reached a level similar to that of the general population. However, the long-term presence of symptoms such as constipation and diarrhea after sphincter-preserving operation continues to affect the Post-QoL of patients and requires timely treatment. In addition, for rectal cancer patients with stage III-IV tumors, the QoL benefit after CSPO is limited. Finally, patients should be fully informed of the negative impact of surgery on their sexual function and sexual enjoyment before CSPO, and postoperative active measures should be taken to promote recovery.

## Data Availability

The raw data supporting the conclusions of this article will be made available by the authors, without undue reservation.

## References

[1] Saito N Ito M Kobayashi A Nishizawa Y Kojima M Nishizawa Y . Long-term outcomes after intersphincteric resection for low-lying rectal cancer. Ann Surg Oncol. (2014) 21:3608–15. doi: 10.1245/s10434-014-3762-y, PMID: 24923221

[2] Sun G Lou Z Zhang H Yu GY Zheng K Gao XH . Retrospective study of the functional and oncological outcomes of conformal sphincter preservation operation in the treatment of very low rectal cancer. Tech Coloproctol. (2020) 24:1025–34. doi: 10.1007/s10151-020-02229-2, PMID: 32361871 PMC7522072

[3] São Julião GP Habr-Gama A Vailati BB Araujo SEA Fernandez LM Perez RO . New strategies in rectal cancer. Surg Clinics North America. (2017) 97:587–604. doi: 10.1016/j.suc.2017.01.008, PMID: 28501249

[4] Bascoul-Mollevi C Gourgou S Borg C Etienne P-L Rio E Rullier E . Neoadjuvant chemotherapy with FOLFIRINOX and preoperative chemoradiotherapy for patients with locally advanced rectal cancer (UNICANCER PRODIGE 23): Health-related quality of life longitudinal analysis. Eur J Cancer. (2023) 186:151–65. doi: 10.1016/j.ejca.2023.03.021, PMID: 37068407

[5] Braun DP Gupta D Grutsch JF Staren ED . Can changes in health related quality of life scores predict survival in stages III and IV colorectal cancer? Health Qual Life Outcomes. (2011) 9:62. doi: 10.1186/1477-7525-9-62, PMID: 21812962 PMC3162879

[6] Sun G Lou Z Zheng K Chen Y Zhang H Wen R . Comparison of functional and oncological outcome of conformal sphincter preservation operation, low anterior resection, and abdominoperineal resection in very low rectal cancer: a retrospective comparative cohort study with propensity score matching. Langenbecks Arch Surg. (2023) 408:208. doi: 10.1007/s00423-023-02925-1, PMID: 37222797

[7] Sun G Zang Y Ding H Chen Y Groothof D Gong H . Comparison of anal function and quality of life after conformal sphincter preservation operation and intersphincteric resection of very low rectal cancer: a multicenter, retrospective, case–control analysis. Tech Coloproctol. (2023) 27:1275–87. doi: 10.1007/s10151-023-02819-w, PMID: 37248369 PMC10638180

[8] Chinese Society of Colorectal Surgery CS of S, Chinese Society of Laparoscopic And Endoscopic Surgery CS of S . Chinese expert consensus on digestive tract reconstruction in mid-low rectal cancer surgery(2021 Edition). Chin J Pract Surg. (2021) 41:1081–9. doi: 10.19538/j.cjps.issn1005-2208.2021.10.01

[9] Araujo RO Vieira FM Victorino AP Torres C Martins I Guaraldi S . Quality of life in a randomized trial comparing two neoadjuvant regimens for locally advanced rectal cancer—INCAGI004. Support Care Cancer. (2022) 30:6557–72. doi: 10.1007/s00520-022-07059-6, PMID: 35486228

[10] Kosmala R Fokas E Flentje M Sauer R Liersch T Graeven U . Quality of life in rectal cancer patients with or without oxaliplatin in the randomised CAO/ARO/AIO-04 phase 3 trial. Eur J Cancer. (2021) 144:281–90. doi: 10.1016/j.ejca.2020.11.029, PMID: 33383348

[11] Zhao H Kanda K . Testing psychometric properties of the standard Chinese version of the european organization for research and treatment of cancer quality of life core questionnaire 30 (EORTC QLQ-c30). J Epidemiol. (2004) 14:193–203. doi: 10.2188/jea.14.193, PMID: 15617393 PMC8784239

[12] Li X Song X Chen Z Li M Lu L Xu Y . Quality of life in rectal cancer patients after radical surgery: A survey of Chinese patients. World J Surg Onc. (2014) 12:161. doi: 10.1186/1477-7819-12-161, PMID: 24886668 PMC4059026

[13] Mercieca-Bebber R Costa DS Norman R Janda M Smith DP Grimison P . The EORTC quality of life questionnaire for cancer patients ( QLQ-c30): Australian general population reference values. Med J Aust. (2019) 210:499–506. doi: 10.5694/mja2.50207, PMID: 31155722

[14] Amin S Joo S Nolte S Yoo HK Patel N Byrnes HF . Health-related quality of life scores of metastatic pancreatic cancer patients responsive to first line chemotherapy compared to newly derived EORTC QLQ-c30 reference values. BMC Cancer. (2022) 22:563. doi: 10.1186/s12885-022-09661-7, PMID: 35596182 PMC9123808

[15] Kaasa S Bjordal K Aaronson N Moum T Wist E Hagen S . The EORTC core quality of life questionnaire (QLQ-c30): Validity and reliability when analysed with patients treated with palliative radiotherapy. Eur J Cancer. (1995) 31:2260–3. doi: 10.1016/0959-8049(95)00296-0, PMID: 8652253

[16] Osoba D Rodrigues G Myles J Zee B Pater J . Interpreting the significance of changes in health-related quality-of-life scores. JCO. (2023) 41:5345–50. doi: 10.1200/JCO.22.02776, PMID: 38056079

[17] Golan T Hammel P Reni M Van Cutsem E Macarulla T Hall MJ . Maintenance olaparib for germline BRCA -mutated metastatic pancreatic cancer. N Engl J Med. (2019) 381:317–27. doi: 10.1056/NEJMoa1903387, PMID: 31157963 PMC6810605

[18] Jørgensen L Paludan-Müller AS Laursen DRT Savović J Boutron I Sterne JAC . Evaluation of the Cochrane tool for assessing risk of bias in randomized clinical trials: overview of published comments and analysis of user practice in Cochrane and non-Cochrane reviews. Syst Rev. (2016) 5:80. doi: 10.1186/s13643-016-0259-8, PMID: 27160280 PMC4862216

[19] Stang A . Critical evaluation of the Newcastle-Ottawa scale for the assessment of the quality of nonrandomized studies in meta-analyses. Eur J Epidemiol. (2010) 25:603–5. doi: 10.1007/s10654-010-9491-z, PMID: 20652370

[20] Wu C Li W Cen D Zhou Q . Is insufficient sleep duration a risk indicator for periodontal disease? a systematic review. Int J Dental Hygiene. (2023) 21:18–27. doi: 10.1111/idh.12633, PMID: 36385732

[21] Borenstein M . How to understand and report heterogeneity in a meta-analysis: The difference between I-squared and prediction intervals. Integr Med Res. (2023) 12:101014. doi: 10.1016/j.imr.2023.101014, PMID: 38938910 PMC11208730

[22] Poelemeijer YQM van der Knaap ETW Marang-van De Mheen PJ Demirkiran A Wiezer MJ Hazebroek EJ . Measuring quality of life in bariatric surgery: A multicentre study. Surg Endosc. (2020) 34:5522–32. doi: 10.1007/s00464-019-07350-4, PMID: 31993820 PMC7644534

[23] Pappou EP Temple LK Patil S Smith JJ Wei IH Nash GM . Quality of life and function after rectal cancer surgery with and without sphincter preservation. Front Oncol. (2022) 12:944843. doi: 10.3389/fonc.2022.944843, PMID: 36353560 PMC9639454

[24] Bohlok A Mercier C Bouazza F Galdon MG Moretti L Donckier V . The burden of low anterior resection syndrome on quality of life in patients with mid or low rectal cancer. Support Care Cancer. (2020) 28:1199–206. doi: 10.1007/s00520-019-04901-2, PMID: 31218414

[25] Chen TY-T Wiltink LM Nout RA Meershoek-Klein Kranenbarg E Laurberg S Marijnen CAM . Bowel function 14 years after preoperative short-course radiotherapy and total mesorectal excision for rectal cancer: Report of a multicenter randomized trial. Clin Colorectal Cancer. (2015) 14:106–14. doi: 10.1016/j.clcc.2014.12.007, PMID: 25677122

[26] Imterat M Gebers G Heitz F Schneider S Ehmann S Welz J . Low anterior resection syndrome and its impact on quality of life of ovarian carcinoma patients: A prospective longitudinal study. Gynecologic Oncol. (2023) 178:96–101. doi: 10.1016/j.ygyno.2023.10.002, PMID: 37839314

[27] Van Heinsbergen M Janssen-Heijnen ML Leijtens JW Slooter GD Konsten JL . Bowel dysfunction after sigmoid resection underestimated: Multicentre study on quality of life after surgery for carcinoma of the rectum and sigmoid. Eur J Surg Oncol. (2018) 44:1261–7. doi: 10.1016/j.ejso.2018.05.003, PMID: 29778617

[28] Ba ZF Yokoyama Y Toth B Rue LW Bland KI Chaudry IH . Gender differences in small intestinal endothelial function: Inhibitory role of androgens. Am J Physiology-Gastrointestinal Liver Physiol. (2004) 286:G452–7. doi: 10.1152/ajpgi.00357.2003, PMID: 14563675

[29] Zafar SY McNeil RB Thomas CM Lathan CS Ayanian JZ Provenzale D . Population-based assessment of cancer survivors’ financial burden and quality of life: A prospective cohort study. JOP. (2015) 11:145–50. doi: 10.1200/JOP.2014.001542, PMID: 25515717 PMC4371118

[30] On behalf of the LARRIS Trial Management Group DL H Cornish J Morris C . Functional outcome following rectal surgery—predisposing factors for low anterior resection syndrome. Int J Colorectal Dis. (2017) 32:691–7. doi: 10.1007/s00384-017-2765-0, PMID: 28130593

[31] Han CJ Gigic B Schneider M Kulu Y Peoples AR Ose J . Risk factors for cancer-related distress in colorectal cancer survivors: One year post surgery. J Cancer Surviv. (2020) 14:305–15. doi: 10.1007/s11764-019-00845-y, PMID: 32166576 PMC7261242

[32] Digennaro R Tondo M Cuccia F Giannini I Pezzolla F Rinaldi M . Coloanal anastomosis or abdominoperineal resection for very low rectal cancer: What will benefit, the surgeon’s pride or the patient’s quality of life? Int J Colorectal Dis. (2013) 28:949–57. doi: 10.1007/s00384-012-1629-x, PMID: 23274737

[33] Silva AL Monteiro PS Sousa JB Vianna AL Oliveira PG . Partners of patients having a permanent colostomy should also receive attention from the healthcare team. Colorectal Dis. (2014) 16:0431–34. doi: 10.1111/codi.12737, PMID: 25104405

[34] Zhao P Yang L Sa Z Wang X . Propriety, empowerment and compromise: Challenges in addressing gender among sex educators in China. Sex Educ. (2020) 20:552–67. doi: 10.1080/14681811.2019.1705779

[35] Cao G Zhang X Wang F Man D Wu L Pan X . Biofeedback combined with percutaneous electrical pudendal nerve stimulation for the treatment of low anterior rectal resection syndrome: a study protocol for a randomized controlled trial. Trials. (2024) 25:440. doi: 10.1186/s13063-024-08300-9, PMID: 38956630 PMC11221096

